# A strategy to protect off-the-shelf cell therapy products using virus-specific T-cells engineered to eliminate alloreactive T-cells

**DOI:** 10.1186/s12967-019-1988-y

**Published:** 2019-07-24

**Authors:** David H. Quach, Luis Becerra-Dominguez, Rayne H. Rouce, Cliona M. Rooney

**Affiliations:** 1 Center for Cell and Gene Therapy, Texas Children’s Hospital, Houston Methodist Hospital and Baylor College of Medicine, 1102 Bates Ave, Suite 1770, Houston, TX 77030 USA; 2 Department of Pediatrics, Baylor College of Medicine, Houston, TX 77030 USA; 3 Department of Pathology and Immunology, Baylor College of Medicine, Houston, TX 77030 USA; 4 Department of Molecular Virology and Immunology, Baylor College of Medicine, Houston, TX 77030 USA

**Keywords:** Allogeneic T-cells, Alloreactive T-cells, Cellular therapy, Graft-vs-host disease, Immunotherapy, Off-the-shelf, Regenerative medicine, Rejection, Tolerance, Virus-specific T-cell

## Abstract

**Background:**

The use of “off-the-shelf” cellular therapy products derived from healthy donors addresses many of the challenges associated with customized cell products. However, the potential of allogeneic cell products to produce graft-versus-host disease (GVHD), and their likely rejection by host alloreactive T-cells are major barriers to their clinical safety and efficacy. We have developed a molecule that when expressed in T-cells, can eliminate alloreactive T-cells and hence can be used to protect cell therapy products from allospecific rejection. Further, expression of this molecule in virus-specific T-cells (VSTs) should virtually eliminate the potential for recipients to develop GVHD.

**Methods:**

To generate a molecule that can mediate killing of cognate alloreactive T-cells, we fused beta-2 microglobulin (B2M), a universal component of all human leukocyte antigen (HLA) class I molecules, to the cytolytic endodomain of the T cell receptor ζ chain, to create a chimeric HLA accessory receptor (CHAR). To determine if CHAR-modified human VSTs could eliminate alloreactive T-cells, we co-cultured them with allogeneic peripheral blood mononuclear cells (PBMC), and assessed proliferation of PBMC-derived alloreactive T-cells and the survival of CHAR-modified VSTs by flow cytometry.

**Results:**

The CHAR was able to transport HLA molecules to the cell surface of Daudi cells, that lack HLA class I expression due to defective B2M expression, illustrating its ability to complex with human HLA class I molecules. Furthermore, VSTs expressing CHAR were protected from allospecific elimination in co-cultures with allogeneic PBMCs compared to unmodified VSTs, and mediated killing of alloreactive T-cells. Unexpectedly, CHAR-modified VSTs eliminated not only alloreactive HLA class I restricted CD8 T-cells, but also alloreactive CD4 T-cells. This beneficial effect resulted from non-specific elimination of activated T-cells. Of note, we confirmed that CHAR-modified VSTs did not affect pathogen-specific T-cells which are essential for protective immunity.

**Conclusions:**

Human T-cells can be genetically modified to eliminate alloreactive T-cells, providing a unique strategy to protect off-the-shelf cell therapy products. Allogeneic cell therapies have already proved effective in treating viral infections in the stem cell transplant setting, and have potential in other fields such as regenerative medicine. A strategy to prevent allograft rejection would greatly increase their efficacy and commercial viability.

**Electronic supplementary material:**

The online version of this article (10.1186/s12967-019-1988-y) contains supplementary material, which is available to authorized users.

## Background

Adoptive cell therapies represent a paradigm shift from conventional drug treatments and offer the potential of treating diseases with greater precision and less toxicity. In particular, T-cell therapies have shown many impressive results. For example, T-cells expressing chimeric antigen receptors (CARs) targeting CD19 have been very effective against B-cell malignancies and virus specific T-cells (VSTs) have shown great promise in treating viral infections in hematopoietic stem cell transplant (HSCT) recipients and patients with Epstein–Barr virus (EBV) associated lymphomas [1–4]. To avoid graft rejection and/or graft-vs-host disease (GVHD), most T-cell therapies are derived directly from the patient or from the stem cell donor in HSCT recipients. This highly personalized strategy has limited wider application due to the time and cost of generating a customized product as well as difficulties in generating therapeutic cell products from some patients with genetic disorders or cancer [5]. If graft rejection and GVHD could be overcome, highly characterized “off-the-shelf” cell products could be derived from healthy donors and dramatically improve the feasibility and availability of cell therapies [6].

The main cellular mediators of both graft rejection and GVHD are alloreactive T-cells that recognize non-self human leukocyte antigen (HLA) molecules on allogeneic cells. To protect allogeneic T-cells from rejection, several groups have eliminated HLA class I expression by knocking out either individual HLA molecules or beta-2 microglobulin (B2M), a universal component of all HLA class I molecules [7, 8]. Although this strategy minimizes T-cell mediated rejection, loss of HLA antigens increases susceptibility to killing by natural killer (NK) cells [9]. The direct elimination of alloreactive T-cells is an alternative approach to graft protection. In 1980, Miller introduced the concept of a “veto cell” that can specifically eliminate a cognate alloreactive T-cell [10, 11]. Although several different cell types, including dendritic cells, NK cells, and T-cells, can demonstrate veto activity, cytotoxic CD8 T-cells are thought to exhibit the strongest effect [12, 13]. Reisner et al. have shown that in murine T-cells this veto effect is independent of T-cell receptor (TCR) ligation and instead is mediated by a Fas–FasL dependent mechanism in which FasL expressed on veto cells binds to Fas on alloreactive T-cells, inducing apoptotic cell death [14–16].

While T-cells may possess an inherent ability to veto alloreactive T-cells without TCR ligation, engagement of the veto cell TCR could initiate a more potent cytolytic effect since it would recruit the more rapidly acting perforin/granzyme pathway [17, 18]. To this end, Margalit et al. constructed a chimera of B2M and the cytolytic domain of the TCR zeta chain [19]. Theoretically, this chimeric B2M/CD3-zeta protein can complex with any HLA class I molecule, and when expressed on an allogeneic T-cell could mediate killing of any engaged alloreactive T-cell. The initial study showed that a murine T-cell hybridoma expressing the B2M/CD3-zeta protein could produce IL-2 when bound by an antibody specific to the murine major histocompatibility complex (MHC) on the T-cell hybridoma, but did not demonstrate veto mediated killing [19]. Subsequent studies focused on autoimmune disease and showed that murine T-cells expressing the B2M/CD3-zeta protein and presenting insulin peptides could reduce progression of diabetes in mice by targeting insulin-specific diabetogenic T-cells [20, 21]. Thus far, however, no studies have evaluated the ability of the B2M/CD3-zeta protein to eliminate alloreactive T-cells or tested this approach in human T-cells.

Preventing allo-rejection overcomes one barrier to off-the-shelf therapy, however, since allogeneic T-cell products may contain alloreactive T-cells that could attack recipient tissues, avoiding GVHD is also essential. To this end, several groups have knocked out the endogenous TCR in T-cells [7, 22, 23], however, complete depletion of TCR positive T-cells may not be feasible and patients infused with less than 1% residual TCR positive T-cells can still develop GVHD [24]. VSTs, by contrast, rarely produce GVHD when infused into allogeneic recipients [25]. Furthermore, allogeneic VSTs that have been banked for use as off-the-shelf therapy have proved safe and effective in treating viral infections in HSCT recipients [26, 27]. Therefore we have used VSTs in our study to avoid the problem of GVHD.

To determine if human T-cells can be engineered to eliminate human alloreactive T-cells, we generated a human version of the B2M/CD3-zeta protein termed the Chimeric HLA Accessory Receptor (CHAR). We found the CHAR could complex with endogenous human HLA class I molecules and carry them to the cell surface. When expressed in VSTs, the CHAR could eliminate alloreactive T-cells in co-cultures with allogeneic peripheral blood mononuclear cells (PBMC) without eliminating pathogen-specific T-cells, and in contrast to unmodified VSTs, were protected from allo-specific elimination. By eliminating alloreactive T-cells, CHAR expressing VSTs could prevent the rejection of allogeneic cell therapy products increasing the persistence of off-the-shelf cell therapies. This strategy could have widespread impact not only on the use of allogeneic cells for the treatment of viral infections and cancer but also on other fields such as regenerative medicine.

## Methods

### Generation of retroviral constructs

The codon optimized CHAR construct was synthesized by GeneArt (Invitrogen, Carlsbad, CA) and cloned into the gamma retroviral vector SFG [28] using In-Fusion cloning (Takara Bio USA, Mountain View, CA). The CHAR sequence consisted of the entire human B2M sequence including the signal peptide, a portion of human HLA-A2 (Uniprot: AA 296-308) to bridge the physical distance between B2M and the cell membrane surface, the transmembrane domain of human CD8 alpha (AA 183-210), and the signaling endodomain of human CD3 zeta chain (AA 52-164). To allow expression of two genes from a single mRNA, a 2A peptide sequence derived from porcine teschovirus-1 with a GSG linker was placed downstream of the CHAR [29]. Downstream of the 2A peptide is the Q8 marker gene that contains a small compact epitope of human CD34 that is recognized by the clinical grade monoclonal antibody QBend10 [30]. This epitope is attached to a human CD8 alpha (CD8a) stalk and transmembrane region (AA 134-222). To limit homologous recombination between the CD8a regions in both Q8 and CHAR constructs, the CD8a region in Q8 was substituted for wildtype CD8a while the CD8a region in the CHAR was codon optimized.

To generate an inducible CHAR we used the Tet-One system from Takara Bio USA (Mountain View, CA) that expresses the two components of the system, the transactivator protein (Tet-On 3G) and the tet-responsive promoter (TRE3GS) in a single plasmid [31]. We inserted our gene product that includes the CHAR, 2A, and Q8 into the Tet-One plasmid downstream of the TRE3GS (Additional file 1: Fig. S1A). As seen in Additional file 1: Fig. S1B, expression of the CHAR from the Tet-One construct resulted in low transduction efficiency in VSTs, consistent with previous reports of low Tet-One transduction in primary T lymphocytes [32]. To improve the transduction efficiency, we made several modified Tet-One constructs (data not shown) and found that inversion of the entire coding sequence between the 5′ LTR and 3′ LTRs (shown in Fig. 2c) resulted in higher transduction (Fig. 2d) compared to the original Tet-One construct (Additional file 1: Fig. S1B). This construct was used for the rest of the study and will be referred to as the inducible CHAR (iCHAR).

### Cell lines

The Daudi cell line was obtained from American Type Culture Collection (ATCC) (Manassas, VA). Daudi cells were maintained in RPMI 1640 media (GE Healthcare Life Sciences, Pittsburgh, PA) supplemented with 10% FBS (GE Healthcare Life Sciences) and 1% GlutaMAX (Thermo Fisher Scientific, Waltham, MA). Cell were grown at 37^o^ C in a humidified atmosphere containing 5% carbon dioxide.

### Generation of T-cells

Peripheral blood mononuclear cells (PBMCs) were isolated from healthy donors after obtaining informed consent under the Institutional Review Board of Baylor College of Medicine and in accordance with the guidelines established by the Declaration of Helsinki. Activated T-cells (ATCs) were generated by plating PBMCs on 24-well plates coated with 1 mg/ml anti-CD3 (OKT3) (ATCC, Manassas, VA) and 1 mg/ml anti-CD28 (BD Biosciences, San Jose, CA). ATCs were maintained in medium with IL-2 (NIH, Bethesda, VA) at 40 IU/ml. Virus specific T-cells (VSTs) were generated from PBMC devoid of CD4 T-cells and NK cells by magnetic column depletion using CD4 and CD56 microbeads (Miltenyi Biotec, Bergisch Gladbach, Germany). Pepmix peptide pools to pp65 (JPT Peptide Technologies, Berlin, Germany) were added to depleted PBMCs (10 ng per 1 × 10^6^ PBMCs) to generate CMV-specific T-cells (CMVSTs). CMVSTs were grown in IL-7 at 10 ng/ml and IL-15 at 10 ng/ml (PeproTech, Rocky Hill, NJ). ATCs and CMVSTs were maintained in medium consisting of a 1:1 mix of RPMI 1640 (GE Healthcare Life Sciences) and Click’s Media (Irvine Scientific, Santa Ana, CA) supplemented with 10% FBS (GE Healthcare Life Sciences) and 1% Glutamax (Thermo Fisher Scientific). Every 2–3 days, T-cells were fed with fresh media containing the respective cytokines. For experiments in which the inducible CHAR was used, certified Tet-Free FBS (Takara Bio USA) was used in place of conventional FBS. Doxycycline (Sigma-Aldrich, St. Louis, MO) was used at 100 ng/ml to induce express of the CHAR.

### Retrovirus production and T-cell transduction

Retroviral supernatants were produced as previously described [33] and plated on non-tissue culture treated 24-well plates pre-coated with RetroNectin (Takara Bio USA). After centrifugation at 2000×*g* for 90 min, retroviral supernatant was removed and CMVSTs from day 4–5 were plated at 0.5 × 10^6^/well. On day 9, CMVSTs were restimulated using a combination of pepmix-pulsed ATCs and a HLA negative costimulatory cell line, K562CS (gift from Carl June), as previously described [34].

### Flow cytometry

The following fluorochrome-conjugated monoclonal antibodies were used in this study: CD3, CD4, CD8, CD19, and IFNγ from Beckman Coulter (Indianapolis, IN); CD71, HLA-A2, and HLA-A, B, C from BioLegend (San Diego, CA); CD95 (Fas) and CD107a from BD Biosciences (San Jose, CA); and CD34 (QBEnd-10) from Abnova (Taipei, Taiwan). Cell viability was assessed using 7-amino actinomycin D (7-AAD) (BD Biosciences) staining. We used the Gallios Flow Cytometer (Beckman Coulter) to acquire flow cytometric data and Kaluza Analysis Software (Beckman Coulter) to analyze data and for graphical representation.

### Co-culture of CMVSTs and allogeneic PBMC

CMVSTs were co-cultured with allogeneic CD56-depleted PBMCs at a 1:2 ratio. Discrimination between CMVSTs and allogeneic PBMCs was determined by HLA-A2 expression. Media contained IL-2 at 20 IU/ml and doxycycline at 100 ng/ml. On days 0, 4 and 8 co-cultures were harvested, stained with antibodies and analyzed by flow cytometry. Countbright Beads (Life Technologies, Carlsbad, CA) were used to assess cell numbers.

### CellTrace Violet proliferation assay

On day 5 after the primary (1st) mixed lymphocyte reaction (MLR) of CMVSTs and allogeneic PBMCs was initiated, all cells within the 1st MLR were stained with CellTrace Violet (Thermo Fisher Scientific, Waltham, MA) at 2.5 μM and then mixed at a 1:1 ratio with PBMCs derived either from the CMVST donor or allogeneic PBMC donor to generate a secondary (2nd) MLR. The 2nd MLR was then harvested after 4–5 days and analyzed by flow cytometry.

### Co-culture of CMVSTs with autologous PBMCs or ATCs

After isolating PBMCs to generate CMVSTs, a portion was cryopreserved for subsequent co-cultures. Thawed autologous PBMCs were rested overnight, and then either these non-activated PBMCs or ATCs, generated by plating the PBMCs on anti-CD3 (ATCC) and anti-CD28 (BD Biosciences) coated plates for 4 h, were labeled with CellTrace Violet (3 μM) and mixed with CMVSTs at a 1:1 ratio. After 4 days in media containing IL-2 at 40 IU/ml, co-cultures were stained with antibodies and analyzed by flow cytometry.

### Knockout of Fas in PBMC and co-culture with CMVST

To knockout Fas in PBMC we used a previously optimized protocol developed by Seki and Rutz [35]. Briefly, we combined 2ul TrueCut Cas9 Protein v2 (Thermo Fisher Scientific, Waltham, MA) with 1 μl each of three single guide RNAs (sgRNA) synthetized by in vitro transcription. CRISPR sgRNA for Fas were designed using the online tool CRISPRscan (https://www.crisprscan.org) and recognized the following target site sequences: GGATTGCTCAACAACCATGCTGG, GATTGCTCAACAACCATGCTGGG, GTGACTGACATCAACTCCAAGGG. The Cas9 and sgRNA complexes were then combined with 2–4 × 10^6^ CD56-depleted PBMC resuspended in 20 μl of buffer solution from the P2 Primary Cell 4D-Nucleofector X Kit S (Lonza, Basel, Switzerland) and put into Nucleofection cuvette strips. Cells were electroporated with the 4D Nucleofector system (4D-Nucleofector Core Unit, AAF-1002B; 4D-Nucleofector X Unit, AAF-1002X) from Lonza (Basel, Switzerland) using the pulse code EH100. After PBMCs were rested overnight at 37 °C, they were mixed at a 10:1 ratio with CMVSTs in media containing IL-2 at 20 IU/ml and doxycycline at 100 ng/ml. Co-cultures were harvested on day 8, stained with antibodies and analyzed by flow cytometry.

### CD107a degranulation assay

CMVSTs, pretreated with doxycycline 1 day prior to induce CHAR expression, were co-cultured with autologous sTCR-ATCs on day 8. Prior to the co-culture, CMVSTs were labeled with CellTrace Violet (0.05 μM) and either pulsed with LML peptide (10 ng per 1 × 10^6^ cells) or a DMSO vehicle control for 1 h. After co-cultures were incubated for 4–5 h in media containing GolgiStop (BD Biosciences) at 1 μl/ml and CD107a antibody (BD Biosciences) at 10 μl/ml, they were stained with additional antibodies and analyzed by flow cytometry.

### Intracellular cytokine staining

CD56-depleted PBMCs were co-cultured with allogeneic iCHAR CMVSTs at a 2:1 ratio or cultured alone for 5 days in media containing IL-2 at 20 IU/ml and doxycycline at 100 ng/ml. Afterward, cells were washed, rested for several hours, and then incubated with pepmixes (500 ng/ml) for adenovirus (Hexon and Penton), CMV (IE1) or EBV (EBNA1, BZLF1, LMP1, and LMP2) overnight in media containing GolgiPlug (BD Biosciences) at 1 μl/ml. The survivin pepmix was used as an irrelevant control. Cells were then labeled with cell surface antibodies, fixed, permeabilized, stained for intracellular IFNγ, and analyzed by flow cytometry. Discrimination between PBMCs and allogeneic CMVSTs was determined by HLA-A2 expression.

### Statistical analysis

Data are presented as mean ± standard error of the mean (SEM) and statistical analysis was performed using GraphPad Prism 5 software (GraphPad Software, Inc.). Paired two-tailed Student *t*-tests were used for comparisons between two groups.

## Results

### A human chimeric HLA accessory receptor (CHAR) can complex with endogenous HLA class I molecules

**Fig. 1:**
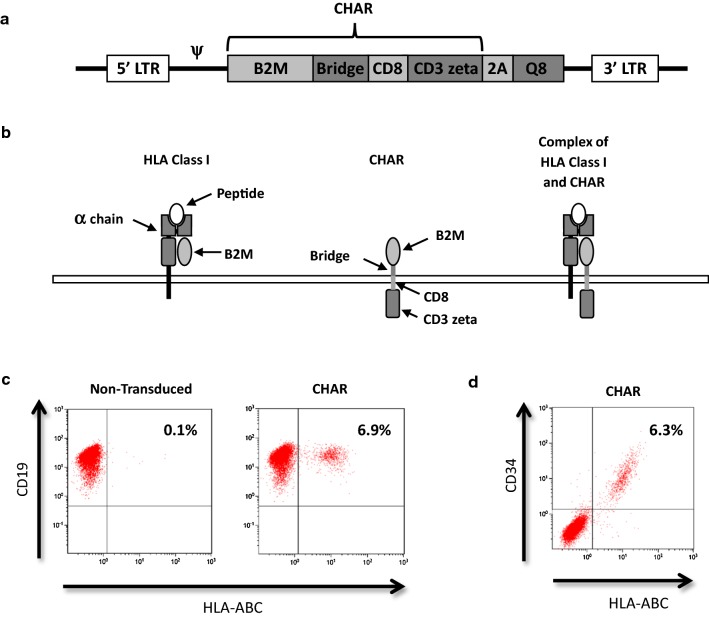
Characterization of Chimeric HLA Accessory Receptor (CHAR). **a** Design of CHAR construct in retrovirus backbone. The Bridge spans the distance between B2M and the cell surface. The Q8 marker contains an epitope from CD34. **b** Schematic of HLA class I molecule (Left) and CHAR (Middle) forming a complex (Right). **c** Daudi cells were transduced with the CHAR construct and stained for expression of HLA class I molecules using a HLA-ABC antibody on Day 3. **d** CHAR transduced Daudi cells were stained for HLA-ABC and CD34 to identify the Q8 marker

To generate a molecule that can mediate the elimination of human alloreactive T-cells by their target cells, human B2M was fused to the human CD3 zeta chain via a short peptide bridge derived from human HLA-A2, allowing the B2M component of the CHAR to align and complex correctly with endogenous HLA class I molecules. We used the transmembrane domain of CD8a because the CD3 zeta transmembrane domain used by Margalit et al. [19] interacts with the endogenous TCR complex and has been reported to be unstable [36, 37]. Figure 1a shows the design of the retroviral vector containing the CHAR and Q8, a compact marker gene containing an epitope of CD34 that is compatible with clinical-grade selection reagents [30]. Figure 1b shows how the CHAR complexes with an HLA class I molecule, and Fig. 1c shows that expression of the CHAR can restore HLA class I expression on Daudi cells that lack surface expression of HLA class I molecules due to B2M deficiency [38]. Further, HLA class I expression on Daudi cells correlated with expression of the Q8 marker as assessed by CD34 staining (Fig. 1d).

### Impaired expansion of virus specific T-cells (VSTs) expressing the CHAR can be overcome using an inducible expression system

We used cytomegalovirus-specific T cells (CMVSTs) as our T-cell platform to express the CHAR, since VSTs carry low risk of causing GVHD in allogeneic recipients [25]. To enrich for CD8 T-cells that are reported to have the strongest veto activity [13], we depleted CD4 T-cells prior to VST generation. We also depleted CD56 positive NK cells that might kill allogeneic KIR-mismatched cells and confuse the analysis of the CHAR activity [39]. CMVSTs were generated by stimulation of PBMCs with an overlapping peptide library representing the immune dominant pp65 antigen of CMV [40]. We transduced the CMVSTs with a retroviral vector expressing the CHAR on day 4–5 and for further expansion, restimulated them with antigen presenting cells (APC) pulsed with CMV peptides on day 9.

**Fig. 2:**
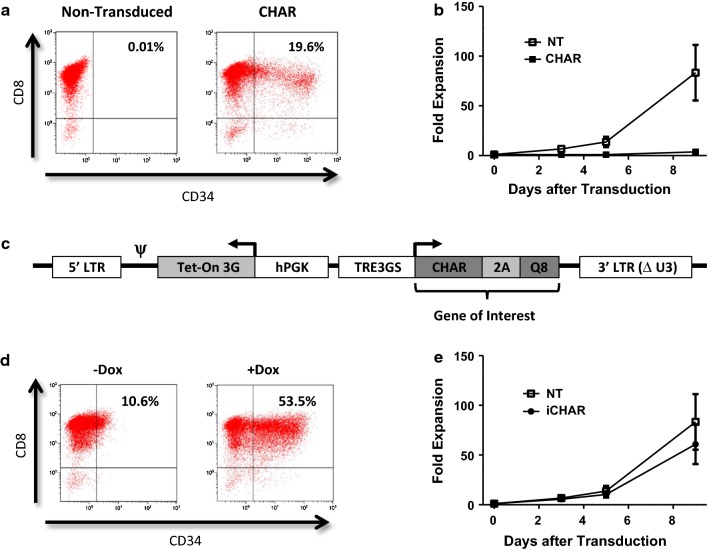
Growth of CMV virus specific T-cells (CMVSTs) expressing the CHAR molecule. **a** CMVSTs were retrovirally transduced with the CHAR construct and stained for CD34 on Day 4 after transduction. **b** Growth curves showing expansion of CMVSTs after transduction with the CHAR molecule (mean ± SEM, n = 3). **c** Design of inducible CHAR construct (iCHAR). **d** CMVSTs expressing the iCHAR were incubated either with Doxycycline or without for 24 h and stained for CD34. Gate set based off of NT conditions. **e** Fold expansion of CMVSTs after transduction with the iCHAR molecule (mean ± SEM, n = 3). *hPGK* human PGK promoter, *TRE3GS* tet response element

Figure 2a shows that CMVSTs can be transduced with the CHAR, however, as seen in Fig. 2b, the transduced CMVSTs showed markedly reduced expansion compared to non-transduced (NT) cells. This was unexpected since impaired growth of murine T-cells expressing similar chimeric B2M/CD3-zeta constructs was not reported [20, 41]. To circumvent this impediment, we used a Tet-On inducible system (Fig. 2c) to generate an inducible CHAR (iCHAR) that is expressed only in the presence of the tetracycline analog doxycycline (Dox), allowing normal expansion of iCHAR CMVSTs when cultured in the absence of Dox. Although, some baseline expression of the CD34 marker was seen even without Dox (Fig. 2d), iCHAR CMVST proliferation was similar to that of NT CMVSTs when grown in the absence of Dox (Fig. 2e).

### iCHAR CMVSTs can limit activation and expansion of alloreactive T-cells and are protected from allospecific elimination

**Fig. 3:**
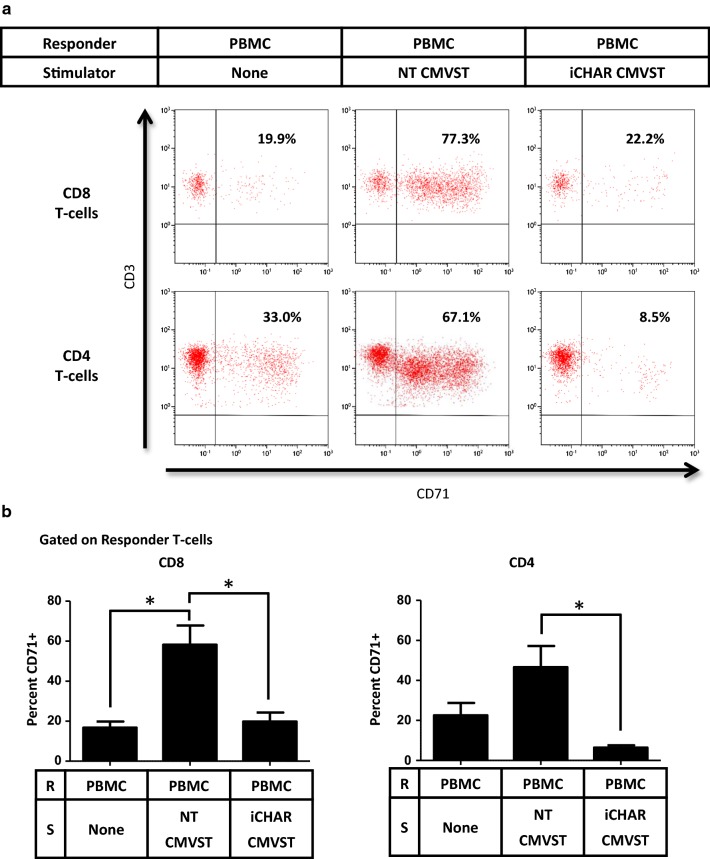
CMVSTs expressing iCHAR can reduce activation of responder alloreactive T-cells. **a** Representative dot plots showing activation of gated responder T-cells assessed by CD71 staining on Day 8. CD8 and CD4 subsets were gated and analyzed separately. **b** Quantification of CD71 + T-cells for both CD8 and CD4 subsets on Day 8 (mean ± SEM, n = 3). Significance was determined by paired two-tailed Student’s *t*-test. *p < 0.05 compared to NT CMVST condition. *R* responder, *S* stimulator

To determine whether iCHAR CMVSTs can eliminate alloreactive T-cells, we established mixed lymphocyte reactions (MLRs) between “stimulator” CMVSTs and “responder” allogeneic PBMCs, in the presence of Dox to induce CHAR expression and low dose IL-2 to support alloreactive T-cell proliferation. By selecting donor pairs differing in HLA-A2 we could distinguish between stimulator CMVSTs and responder PBMCs using an HLA-A2 antibody. To measure alloreactive T-cell activity, we examined expression of the activation marker CD71, which is upregulated in response to alloantigens [42], on PBMC-derived responder T-cells after co-culture with CMVSTs. Figure 3a shows CD71 expression on gated responder CD8 and CD4 T-cell subsets on day 8 after co-culture with CMVSTs in a representative experiment. PBMCs cultured alone showed some baseline CD71 expression, likely due to the presence of IL-2, while PBMCs cultured with non-transduced (NT) CMVSTs significantly upregulated CD71 in both CD8 and CD4 responder T-cells subsets (Fig. 3b). By contrast, after culturing with iCHAR CMVSTs, significantly less CD71 expression was observed in both CD8 and CD4 responder T-cell subsets (Fig. 3b).

**Fig. 4:**
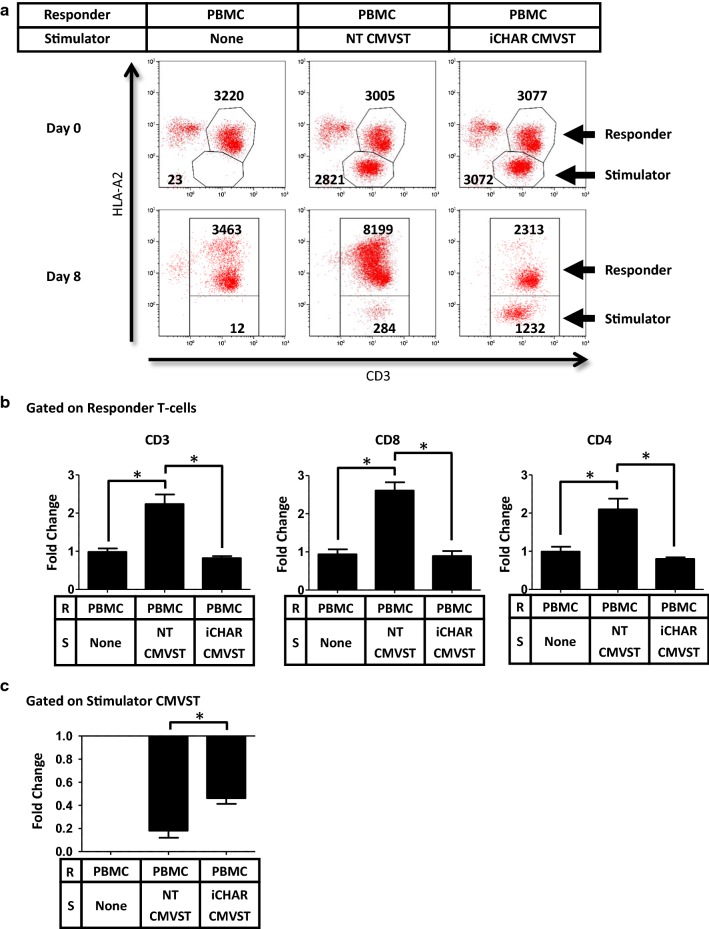
CMVSTs expressing iCHAR can reduce expansion of responder alloreactive T-cells. **a** Responder PBMCs were co-culture with stimulator CMVSTs and flow cytometry analysis was perform on Day 0 and Day 8. Representative dot plots of co-cultures showing cell counts assessed by counting beads. HLA-A2 staining distinguishes between responder and stimulator cells. **b** Calculated fold change of cell counts from Day 0 to Day 8 of gated responder T-cells (mean ± SEM, n = 3). **c** Calculated fold change from Day 0 to Day 8 of gated stimulator CMVSTs (mean ± SEM, n = 3). Significance was determined by paired two-tailed Student’s *t*-test. *p < 0.05 compared to NT CMVST condition. *R* responder, *S* stimulator

The proliferation/survival of responder T-cells and stimulator CMVSTs were quantified as fold change from day 0 to day 8 using counting beads in the flow cytometry assays. Figure 4a shows a representative analysis of responder T-cells and stimulator CMVSTs discriminated based on HLA-A2 expression. PBMCs cultured alone maintained their T-cell numbers by day 8. However, when cultured with NT CMVSTs, responder T-cells within the PBMCs proliferated extensively (Fig. 4b), corresponding to a decrease in CMVSTs (Fig. 4c), suggesting that alloreactive T-cells in the PBMCs proliferated and killed the CMVSTs. By contrast, in the presence of iCHAR CMVSTs, responder T-cell proliferation was significantly lower (Fig. 4b), while iCHAR CMVSTs were preserved (Fig. 4c), indicating that the CHAR can protect against allospecific killing and prevent alloreactive T-cell expansion. Unexpectedly, both CD8 and CD4 T-cells within the PBMCs were inhibited (Fig. 4b), which while beneficial, was surprising, since CD4 T-cells are HLA class II restricted and theoretically should not interact with the CHAR/HLA class I complex.

### Secondary mixed lymphocyte reactions (MLRs) suggest that alloreactive T-cells are eliminated by iCHAR CMVSTs

**Fig. 5:**
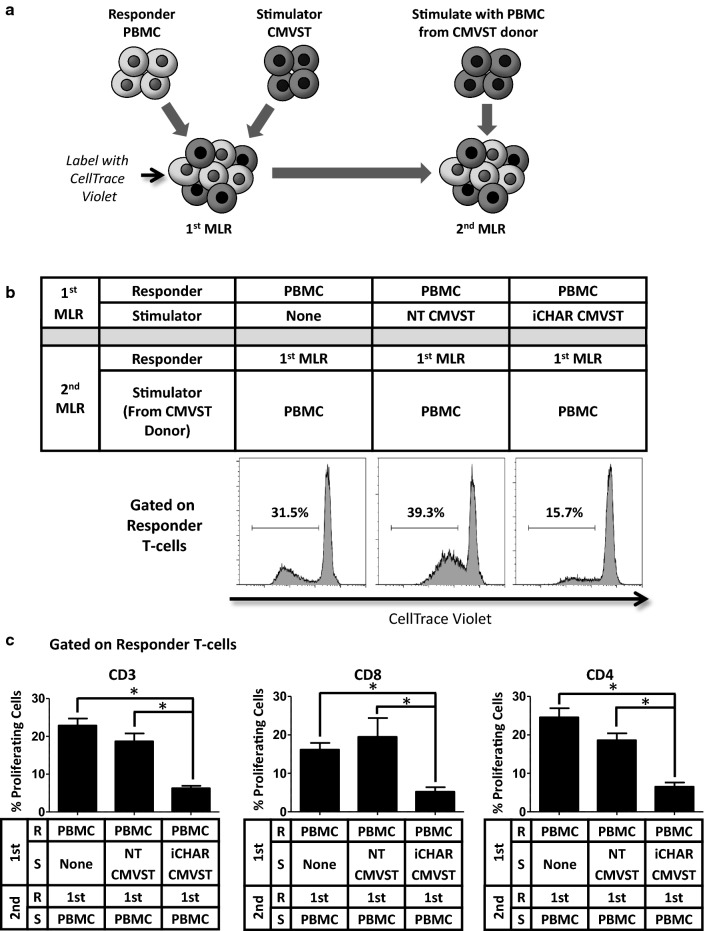
CMVSTs expressing iCHAR reduce responder alloreactive T-cells as assessed by secondary MLR. **a** Primary (1st) MLRs were labeled with CellTrace Violet 5 days after setup and then restimulated with PBMCs derived from the stimulator CMVST donor to generate secondary (2nd) MLRs. 2nd MLRs were analyzed by flow cytometry after 4–5 days. **b** Representative histogram plots showing CellTrace Violet staining on gated responder T-cells. Dilution of CellTrace Violet indicates cell proliferation. **c** Quantification of low CellTrace Violet stained responder T-cells to assess proliferation (mean ± SEM, n = 3). Significance was determined by paired two-tailed Student’s *t*-test. *p < 0.05 compared to iCHAR CMVST condition. *R* responder, *S* stimulator

Primary (1st) MLRs showed that iCHAR CMVST can limit the expansion of alloreactive T-cells within allogeneic PBMCs. To determine if the alloreactive T-cells were eliminated or just inhibited, we harvested the 1st MLR on day 5, labeled it with CellTrace Violet to allow assessment of subsequent proliferation, and then restimulated with fresh PBMCs derived from the CMVST donor for 4–5 days to generate a secondary (2nd) MLR (Fig. 5a). PBMCs that were cultured alone during the 1st MLR proliferated in response to PBMCs from the allogeneic CMVST donor, representing an unprimed allospecific response (Fig. 5b, left panel). PBMCs primed with NT CMVST in the 1st MLR, show a secondary response to the PBMCs of the CMVST donor in the 2nd MLR (Fig. 5b, middle panel). However, PBMCs cultured with iCHAR CMVSTs in the 1st MLR showed a greatly reduced frequency of proliferating T-cells in the 2nd MLR compared to the other two conditions (Fig. 5b, right panel). To control for potential “feeder” effects of co-culture with PBMCs, we setup 2nd MLRs in which responder T-cells were cultured with autologous PBMCs (Additional file 2: Fig. S2) and we subtracted this background proliferation to calculate the percent of proliferation resulting exclusively from the allospecific reaction. Figure 5c shows background subtracted values confirming a significant reduction in the frequency of proliferating alloreactive T-cells in the 2nd MLR for the iCHAR CMVST containing condition compared to the other two conditions for both the CD8 and CD4 T-cell subsets. These results suggest that alloreactive T-cells were indeed eliminated during the 1st MLR.

### iCHAR CMVSTs can non-specifically inhibit activated T-cells

**Fig. 6:**
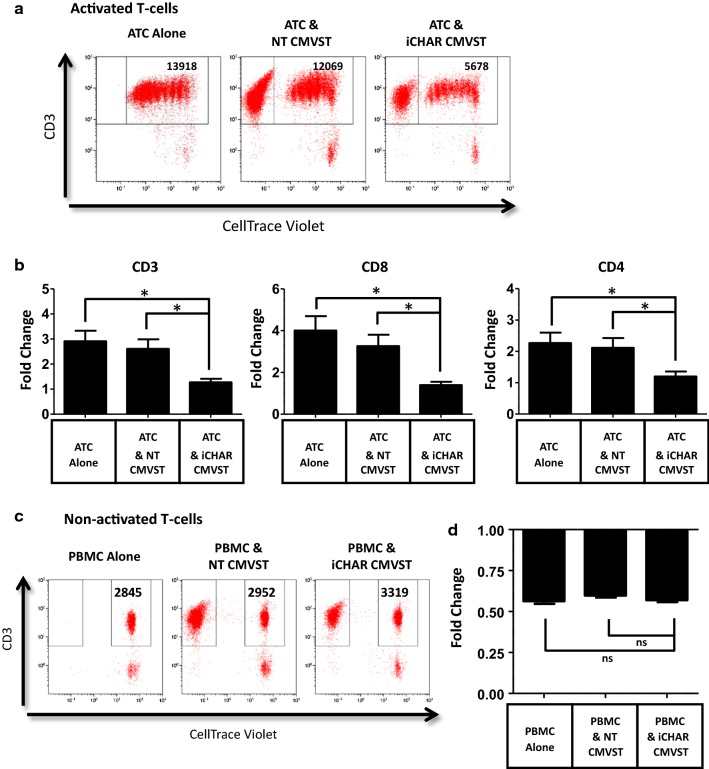
CMVSTs expressing iCHAR reduce expansion of activated T-cells but do not affect resting non-activated T-cells. **a** PBMCs labeled with CellTrace Violet were activated with antibodies to CD3 and CD28 for 4 h and then co-cultured with CMVSTs for 4 days. Representative dot plots of co-cultures from Day 4 showing cell counts of activated T-cells (ATC) assessed by counting beads. **b** Calculated fold change of gated ATC (CD3 + and CellTrace Violet+) from Day 0 to Day 4 (mean ± SEM, n = 3). **c** Non-activated PBMCs labeled with CellTrace Violet were co-cultured with CMVST for 4 days. Representative dot plots of co-cultures from Day 4 showing cell counts of non-activated T-cells. **d** Calculated fold change of gated non-activated T-cells (CD3+ and CellTrace Violet+) from Day 0 to Day 4 (mean ± SEM, n = 3). Significance was determined by paired two-tailed Student’s *t*-test. *p < 0.05 compared to iCHAR CMVST condition. *ns* not significant

As noted, alloreactive CD4 T-cells were also eliminated by the CHAR. One potential explanation for this observation is that the CHAR mediates non-specific killing of activated T-cells. This may also explain the impaired expansion of CHAR CMVSTs seen in Fig. 2b. To test this theory, we co-cultured iCHAR CMVSTs with autologous CD3 and CD28 antibody-activated T-cells (ATCs) labeled with CellTrace Violet and calculated ATC expansion during a 4 day co-culture. iCHAR CMVSTs significantly decreased the expansion of both autologous CD8 and CD4 ATCs compared to ATCs cultured alone or with NT CMVST (Fig. 6a, b). However, proliferation was not completely inhibited since dilution of CellTrace Violet was still observed (Fig. 6a). Notably, iCHAR CMVSTs did not kill resting T-cells, since no significant reduction of autologous PBMCs was observed after culturing with iCHAR CMVSTs, compared to when PBMCs were cultured alone or with NT CMVSTs (Fig. 6c, d).

The non-specific killing of activated T-cells may be mediated by Fas–FasL interactions. However, we observed no differences in the frequency or mean fluorescence intensity of Fas expression in NT CMVST, iCHAR CMVST or in the responding alloreactive T-cells after co-culture (Data not shown). We next sought to assess whether differential expression of FasL on CMVSTs could explain the difference in killing of activated T-cells by iCHAR CMVSTs compared to NT CMVSTs. Unfortunately, as reported by other groups, we found detection of FasL to be difficult and unreliable [43–45]. To circumvent this issue, we knocked out Fas expression on allogeneic PBMCs prior to co-culture with CMVSTs using CRISPR Cas9. We followed the protocol developed by Seki and Rutz [35] in which PBMC are nucleofected with Cas9 protein and single guide RNAs (sgRNA). As shown in Additional file 3: Fig S3A, we achieved greater than 90% knockout efficiency of Fas expression in PBMC nucleofected with Fas-directed sgRNA. We then evaluated the ability of NT CMVST or iCHAR CMVST to eliminate Fas-deficient CD4 and CD8 alloreactive T-cells in co-cultures. As shown in Additional file 3: Fig S3B and S3C, iCHAR CMVST efficiently eliminated activated CD4 and CD8 T-cells compared to NT CMVST. These results are similar to those shown in Fig. 3a, b, indicating that the Fas-FasL pathway is not the major mediator of the non-specific killing of activated CD4 T-cells by iCHAR CMVSTs.

### iCHAR CMVSTs degranulate when targeted by a cognate TCR

**Fig. 7:**
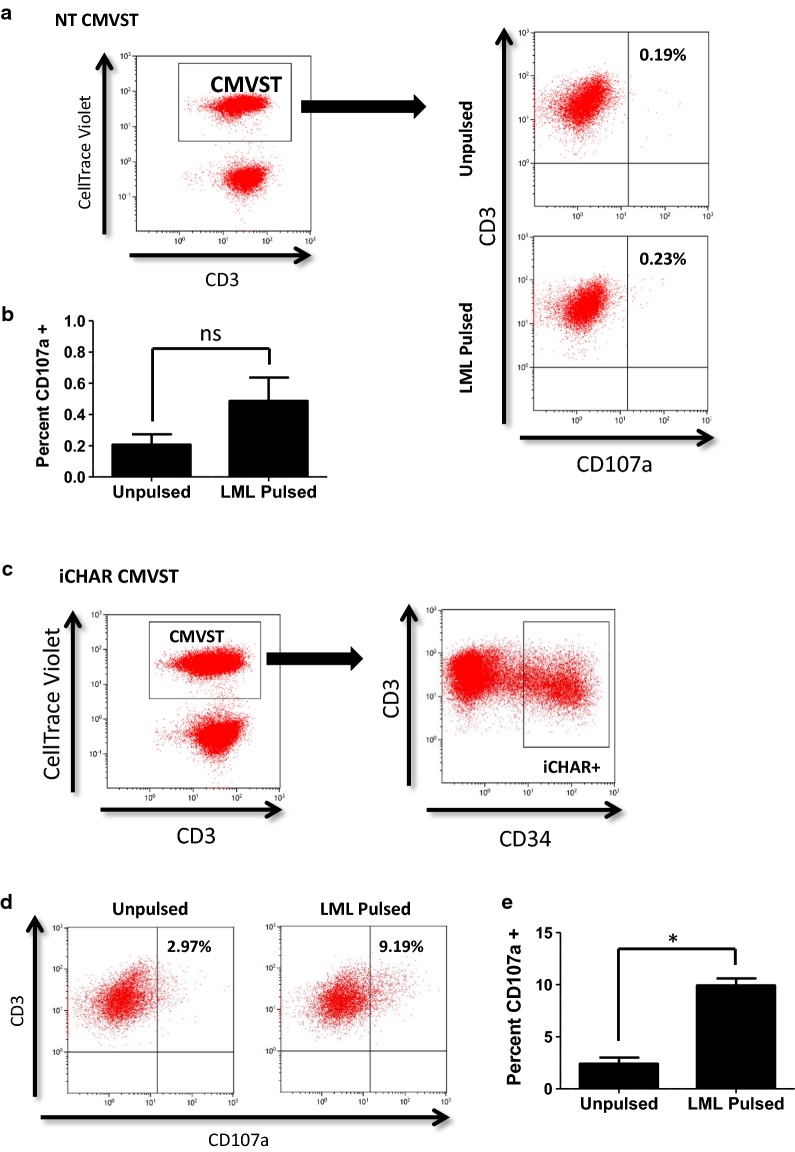
CMVST expressing iCHAR can degranulate when specifically targeted by cognate TCR. **a** CMVST that were unpulsed or LML pulsed were co-cultured with autologous T-cells expressing sTCR for 4 h and then stained for CD107a. Representative dot plots showing gating of CellTrace Violet + NT CMVSTs (Left) and CD107a expression for NT CMVSTs (Right). **b** Quantification showing percent of gated NT CMVSTs expressing CD107a (mean ± SEM, n = 3). **c** Gating for the iCHAR CMVST condition was first done on CellTrace Violet + CMVSTs and then on high iCHAR + T-cells. **d** Representative dot plots showing CD107a expression from gated iCHAR + CMVSTs. **e** Quantification showing percent of gated iCHAR + CMVSTs expressing CD107a (mean ± SEM, n = 3). Significance was determined by paired two-tailed Student’s *t*-test. *p < 0.05. *ns* not significant

Since CHAR-mediated T-cell inhibition was not restricted to alloreactive T-cells, we designed an experiment to determine if the CHAR functions as designed, and is activated when engaged by a cognate TCR. To this end, we used a surrogate T-cell population with an easily identifiable recombinant HLA-A2 restricted survivin-specific TCR (sTCR) [46] that would recognize autologous CMVSTs only if presenting the cognate survivin peptide, LMLGEFLKL (LML). This approach allowed us to determine if iCHAR CMVSTs presenting LML can degranulate when targeted by the cognate sTCR. We labeled unpulsed or LML-pulsed, NT or iCHAR CMVSTs with CellTrace Violet, then measured their degranulation after a 4 h co-culture with autologous ATCs expressing sTCR (sTCR-ATCs), using a CD107 detection assay. NT CMVSTs show little CD107a expression whether or not they were pulsed with the LML peptide (illustrated in Fig. 7a and quantified in Fig. 7b). By contrast, gated iCHAR + CMVSTs (Fig. 7c) co-cultured with sTCR-ATCs expressed significantly more CD107a when they were pulsed with the LML peptide (Fig. 7d and e). This confirms that the CHAR can be activated directly by a cognate TCR. Of note, unpulsed iCHAR CMVSTs (Fig. 7c, left plot) showed higher baseline CD107a expression compared to NT CMVSTs (Fig. 7a), likely due to self-recognition of low level T-cell activation leading to background degranulation. Confirming the specificity of the sTCR to the LML peptide, sTCR-ATCs expressed CD107a only when CMVSTs were pulsed with the LML peptide (Additional file 4: Fig. S4A and B).

### iCHAR CMVSTs do not significantly reduce the frequency of VSTs in allogeneic PBMCs

**Fig. 8:**
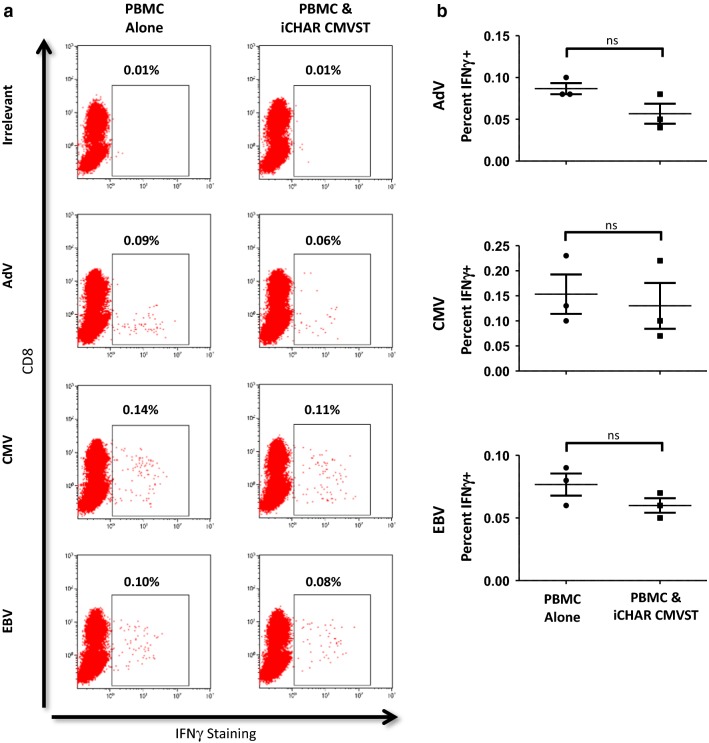
CMVSTs expressing iCHAR do not lower frequency of virus specific T-cells present in allogeneic PBMCs. **a** Allogeneic PBMCs were either cultured alone or with iCHAR CMVST for 5 days and then co-cultures were pulsed with various viral pepmixes. IFNy staining was performed after overnight incubation with viral pepmixes. Shown are representative dot plots of IFNy expression from gated T-cells in the PBMCs after stimulation with Adenovirus (AdV), CMV or EBV pepmixes. Irrelevant represents pepmix for Survivin which should not elicit a response in PBMCs. **b** Quantification of IFNy expression from gated T-cells in PBMCs. Values for each virus were calculated by subtracting from Irrelevant control (mean ± SEM, n = 3). Significance was determined by paired two-tailed Student’s *t*-test. *ns* not significant

If iCHAR VSTs are to be infused into immune competent recipients, it is important that they do not eliminate endogenous pathogen-specific T-cells. We therefore co-cultured iCHAR CMVSTs with allogeneic PBMCs for 5 days, then assessed the frequency of VSTs within the remaining PBMCs by measuring interferon gamma (IFNγ) production in response to overlapping peptide libraries representing immunodominant antigens from several different viruses (Adenovirus, CMV and EBV) using intracellular cytokine staining. We found that the frequency of IFNγ-secreting VSTs in allogeneic PBMCs was not significantly decreased in the presence of iCHAR CMVSTs (Fig. 8a, b).

## Discussion

In this study, we show that human VSTs engineered to express a humanized iCHAR can limit the activation and expansion of alloreactive T-cells via the perforin/granzyme pathway and notably do not affect pathogen-specific memory T-cells, such as VSTs, which should spare recipients from general immune dysfunction. To the best of our knowledge, this is the first study to demonstrate that primary human T-cells can be engineered to enhance their veto activity, potentially allowing iCHAR VSTs to be used as a platform for future off-the-shelf allogeneic T-cell therapies.

Unexpectedly, the human CHAR was not exclusively specific for alloreactive HLA class I-restricted CD8 T-cells but also targeted alloreactive CD4 T cells and irrelevant activated T-cells, a characteristic that likely contributed to the observed fratricide of CHAR VSTs. The mechanisms underlying these findings still remain unclear. We excluded a role for the Fas-FasL pathway, since iCHAR CMVSTs were still able to eliminate activated CD4 alloreactive T-cells lacking Fas. The exact mechanism responsible for these effects is an interesting avenue for future studies, and may result from phenotypic changes that occur in response to T-cell activation [47, 48]. For example, upregulation of killer cell immunoglobulin-like receptors (KIRs) that bind HLA class I molecules [49] may render activated T-cells susceptible to killing by the CHAR. We are currently investigating this and other possible mechanisms that can explain the killing of activated T-cells as well as CHAR-mediated fratricide.

This unanticipated targeting of activated T-cells by CHAR VSTs was fortuitous, since it resulted in killing of alloreactive CD4 T-cells that can also mediate rejection of allografts [50, 51]. However, targeting all activated T-cells could be detrimental if left unchecked, since pathogen-specific T-cell responses could also be impaired. Fortunately, the iCHAR is drug inducible, so that withdrawal of drug after the elimination of alloreactive T-cells should downregulate the iCHAR in VSTs allowing them to respond normally to viral infections and provide undisturbed protective immunity.

A barrier to the clinical use of inducible expression systems in cell therapy is their frequent reliance on xenogeneic and hence immunogenic components, such as our bacterial derived Tet transactivator protein, which could induce immune mediated elimination of engineered therapeutic cells [32, 52]. As our iCHAR VSTs have veto ability, any T-cell that recognized Tet transactivator-derived epitopes would be eliminated. Thus, combining the veto ability of our CHAR VSTs with an inducible expression system improves the safety of CHAR VSTs while negating the immunogenicity of the inducible system. This ability to eliminate T-cells that recognize immunogenic proteins could have widespread implications in the field of synthetic biology in which cells can be endowed with sophisticated capabilities but often use components derived from viruses and bacteria to achieve high specificity and potency [53–55]. Such a strategy to tolerize patients to immunogenic foreign proteins could pave the way for clinical translation of synthetic biology.

Reisner and colleagues have shown that unmodified murine T-cells can veto/eliminate alloreactive T-cells leading to allografts acceptance in murine models [56, 57]. To our knowledge however, effective translation of this work to humans has yet to be demonstrated. In our hands, unmodified activated human T-cells did not prevent the expansion of human alloreactive T-cells and only after CHAR transduction did they develop significant veto activity. These discrepancies in veto efficacy of unmodified T-cells may be due to differences in the veto cell types examined, the assays used to assess veto activity, or to inherent disparities in veto mechanisms between mouse and human cells. Given that the immune systems of mice and humans can differ in significant ways [58, 59], characterizing alloreactivity and tolerance mechanisms in human cells may be more appropriate for the evaluation of therapies that can translate effectively to the clinic.

Elimination of alloreactive T-cells by iCHAR VSTs could tolerize recipients to allow protection not only of iCHAR VSTs but also other cell therapy products that are matched to the iCHAR VST donor. As direct effectors, rejection resistant allogeneic iCHAR VSTs could be used to treat viral infections [26, 60] and malignancies [61]. Alternatively, iCHAR VSTs transduced with CARs [62, 63] could be used as off-the-shelf products for a range of malignancies. However, since iCHAR VSTs may not optimally perform the function of a veto cell and effector cell at the same time, it may be more effective to use iCHAR VSTs to protect subsequently infused therapeutic T-cell products derived from the same donor.

Protecting allogeneic cell therapy products beyond T-cells could significantly impact many other fields such as regenerative medicine in which immune rejection could be a critical barrier to long-term therapy [64, 65]. Similarly, in transplantation biology, eliminating alloreactive T-cells to induce lasting tolerance could prevent rejection of both solid organ and stem cell transplants without the use of immunosuppressants that are associated with long-term toxicities [12, 66]. Since alloreactive T-cells also mediate GVHD [67], iCHAR VSTs derived from the patient could be used to prevent or treat GVHD by eliminating anti-host alloreactive T-cells present in the graft. In addition, patient-derived iCHAR VSTs could potentially prevent or treat autoimmune diseases such as type 1 diabetes if they could be further modified to eliminate self-reactive T-cells like diabetogenic T-cells, as demonstrated in mice by Wong and colleagues [20, 41].

## Conclusions

This proof-of-concept study shows that human T-cells expressing an iCHAR can eliminate alloreactive T-cells and provides platforms both to tolerize recipients to allogeneic off-the-shelf cell therapy products and to protect recipients from GVHD. Successful translation of this approach to the clinic could have significant impact not only on cell therapies, but on regenerative medicine, transplantation, and autoimmunity.

## Additional files

**Additional file 1: Fig. S1.** Characterization of initial inducible CHAR using Clontech’s original Tet-One plasmid. (A) Design of initial inducible CHAR construct with the 3′LTR upstream and 5′LTR downstream of the CHAR construct. (B) CHAR expressing CMVSTs were incubated either with Doxycycline or without for 24 h and stained for CD34. Gate set based off of NT conditions.

**Additional file 2: Fig. S2.** Assessing background proliferation resulting from PBMC in the secondary (2nd) MLR. (A) Primary (1st) MLRs were restimulated with autologous PBMC derived from the responder donor to assess background proliferation resulting from non-specific growth promoting effects of PBMCs alone. Shown are representative histogram plots of CellTrace Violet staining of gated responder T-cells.

**Additional file 3: Fig. S3.** CMVSTs expressing iCHAR can still reduce activation of responder alloreactive T-cells that lack Fas expression. (A) Knockout of Fas in allogeneic PBMC using CRISPR technology. Freshly isolated PBMC were nucleofected with Cas9 and single guide RNAs (sgRNA) to Fas and rested overnight. PBMC were then co-cultured with CMVSTs and Fas expression on gated responder T-cells was measured on Day 8. (B) CMVSTs were co-cultured with PBMC that were knocked out for Fas. On Day 8, activation of gated responder T-cells was assessed by CD71 staining. CD8 and CD4 subsets were gated and analyzed separately. (C) Quantification of CD71+ T-cells for both CD8 and CD4 subsets on Day 8 (mean ± SEM, n = 3). Of note, the level of activation of allogeneic PBMCs that are knocked out for Fas was lower compared to when unmodified, which is likely due to the non-specific toxicity associated with electroporation and knockout impairing the allo-reaction. Significance was determined by paired two-tailed Student’s *t*-test. *p < 0.05 compared to NT CMVST condition. R = Responder, S = Stimulator.

**Additional file 4: Fig. S4.** T-cells expressing sTCR respond specifically to LML pulsed targets. (A) Shown is the gating strategy where first we gated on CellTrace Violet negative T-cells and then on sTCR expressing T-cells. (B) “Responder” sTCR T-cells show CD107a degranulation only when CMVSTs were pulsed with the LML peptide. Shown are representative flow plots for the NT CMVSTs condition.

## Data Availability

Data and materials generated during the current study are available upon reasonable request from the corresponding author.

## Abbreviations

7-AAD: 7-amino actinomycin D
AdV: adenovirus
APC: antigen presenting cells
ATC: activated T-cells
ATCC: American Type Culture Collection
B2M: beta-2 microglobulin
CAR: chimeric antigen receptor
CHAR: chimeric HLA accessory receptor
CMV: cytomegalovirus
CMVST: cytomegalovirus-specific T-cells
Dox: doxycycline
EBV: Epstein–Barr virus
GVHD: graft-vs-host disease
HLA: human leukocyte antigen
HSCT: hematopoietic stem cell transplant
iCHAR: inducible chimeric HLA accessory receptor
IFNγ: interferon gamma
KIR: killer cell immunoglobulin-like receptor
LML: LMLGEFLKL peptide
MHC: major histocompatibility complex
MLR: mixed lymphocytes reactions
NT: non-transduced
PBMC: peripheral blood mononuclear cell
sgRNA: single guide RNA
sTCR: survivin-specific TCR
TCR: T-cell receptor
VST: virus specific T-cell
