# Supplementary figures and images for: A strategy to protect off-the-shelf cell therapy products using virus-specific T-cells engineered to eliminate alloreactive T-cells
Source: J Transl Med. 2019 Jul 24;17:240. doi: 10.1186/s12967-019-1988-y (PMC6657103; doi:10.1186/s12967-019-1988-y)

**A**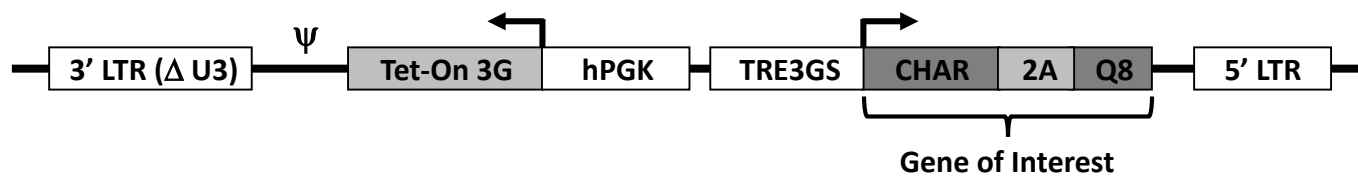**B**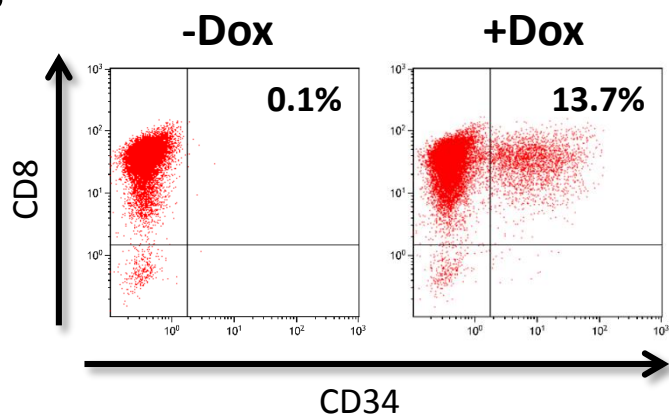

Supplement: Supplementary file 1 — Additional file 1: Fig. S1. Characterization of initial inducible CHAR using Clontech’s original Tet-One plasmid. (A) Design of initial inducible CHAR construct with the 3′LTR upstream and 5′LTR downstream of the CHAR construct. (B) CHAR expressing CMVSTs were incubated either with Doxycycline or without for 24 h and stained for CD34. Gate set based off of NT conditions. [file 12967_2019_1988_MOESM1_ESM.pdf]

**A**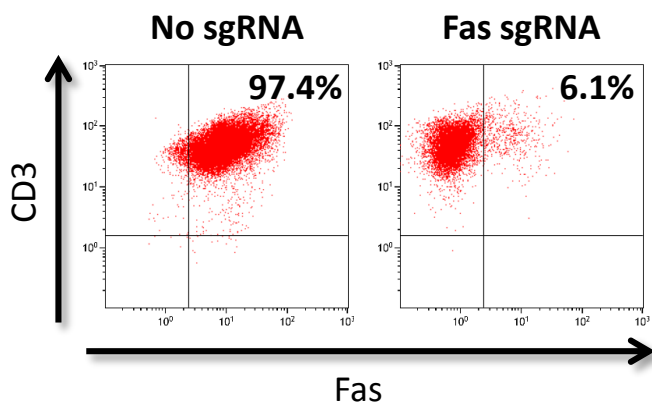**B**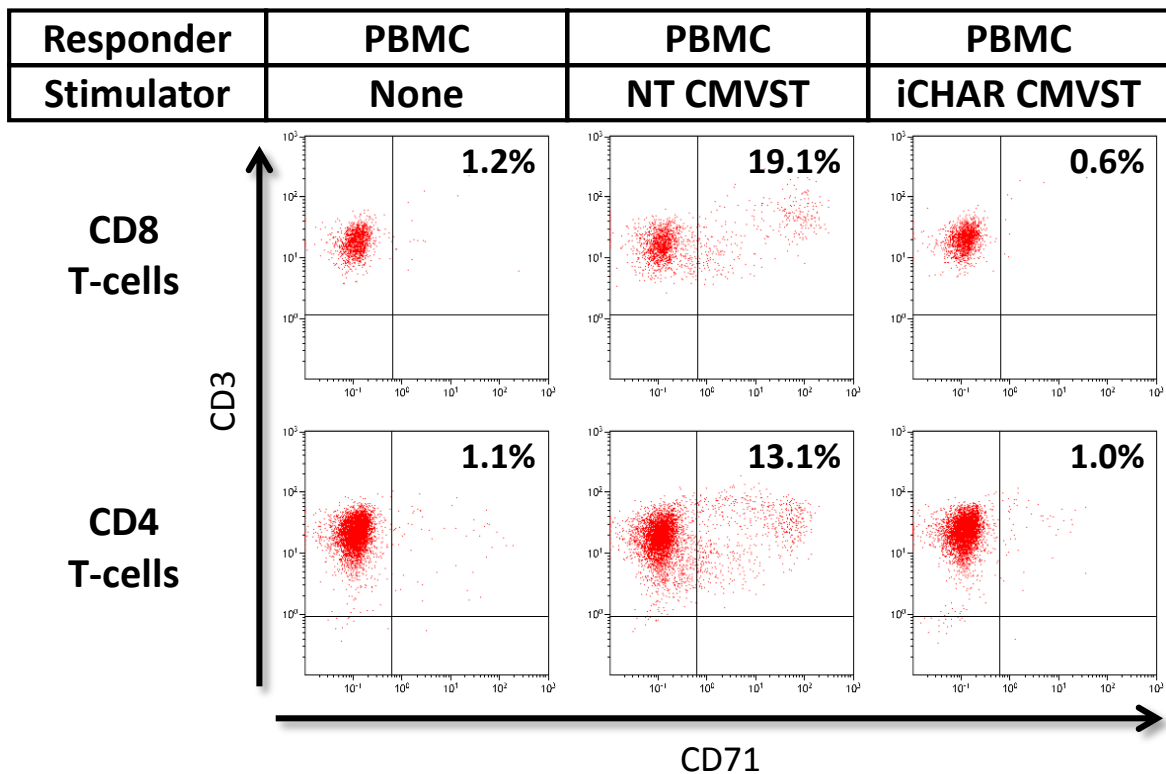**C**

Gated on Responder T-cells

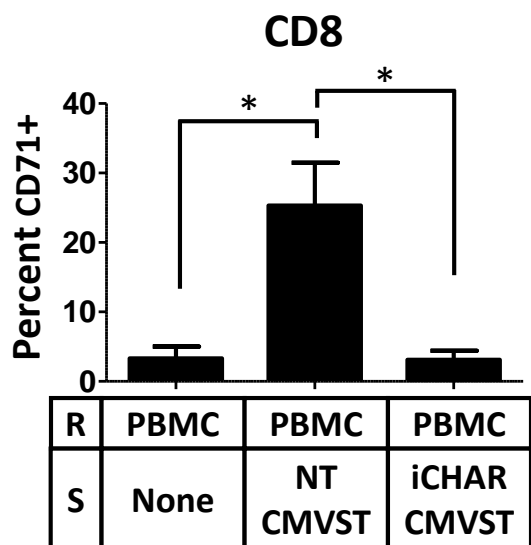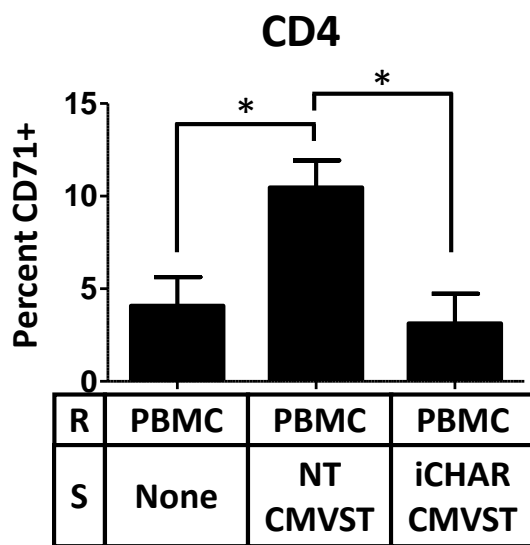

Supplement: Supplementary file 3 — Additional file 3: Fig. S3. CMVSTs expressing iCHAR can still reduce activation of responder alloreactive T-cells that lack Fas expression. (A) Knockout of Fas in allogeneic PBMC using CRISPR technology. Freshly isolated PBMC were nucleofected with Cas9 and single guide RNAs (sgRNA) to Fas and rested overnight. PBMC were then co-cultured with CMVSTs and Fas expression on gated responder T-cells was measured on Day 8. (B) CMVSTs were co-cultured with PBMC that were knocked out for Fas. On Day 8, activation of gated responder T-cells was assessed by CD71 staining. CD8 and CD4 subsets were gated and analyzed separately. (C) Quantification of CD71+ T-cells for both CD8 and CD4 subsets on Day 8 (mean ± SEM, n = 3). Of note, the level of activation of allogeneic PBMCs that are knocked out for Fas was lower compared to when unmodified, which is likely due to the non-specific toxicity associated with electroporation and knockout impairing the allo-reaction. Significance was determined by paired two-tailed Student’s t-test. *p < 0.05 compared to NT CMVST condition. R = Responder, S = Stimulator. [file 12967_2019_1988_MOESM3_ESM.pdf]

**A**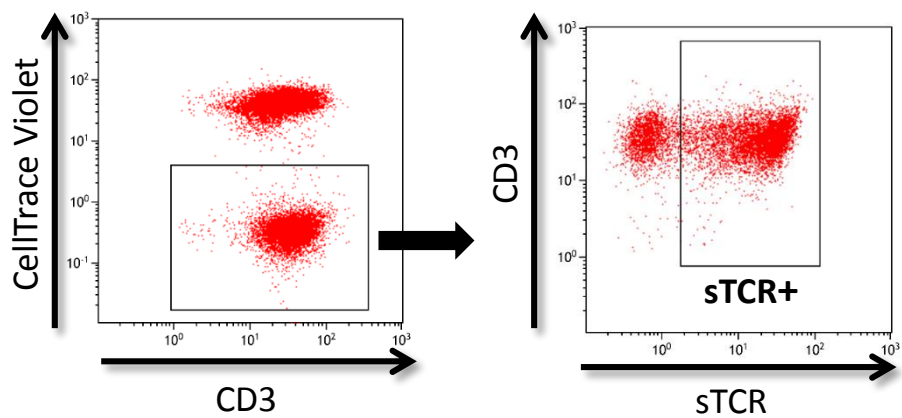**B**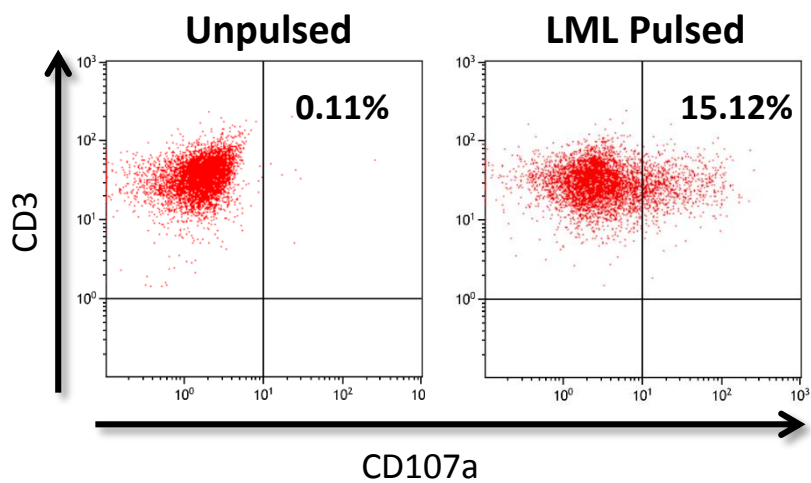

Supplement: Supplementary file 4 — Additional file 4: Fig. S4. T-cells expressing sTCR respond specifically to LML pulsed targets. (A) Shown is the gating strategy where first we gated on CellTrace Violet negative T-cells and then on sTCR expressing T-cells. (B) “Responder” sTCR T-cells show CD107a degranulation only when CMVSTs were pulsed with the LML peptide. Shown are representative flow plots for the NT CMVSTs condition. [file 12967_2019_1988_MOESM4_ESM.pdf]
