# Supplementary material for: A strategy to protect off-the-shelf cell therapy products using virus-specific T-cells engineered to eliminate alloreactive T-cells
Source: J Transl Med. 2019 Jul 24;17:240. doi: 10.1186/s12967-019-1988-y (PMC6657103; doi:10.1186/s12967-019-1988-y)

1st MLR	Responder	PBMC	PBMC	PBMC
	Stimulator	None	NT CMVST	iCHAR CMVST
2nd MLR	Responder	1st MLR	1st MLR	1st MLR
	Stimulator (Responder Donor)	PBMC	PBMC	PBMC

Gated on Responder T-cells

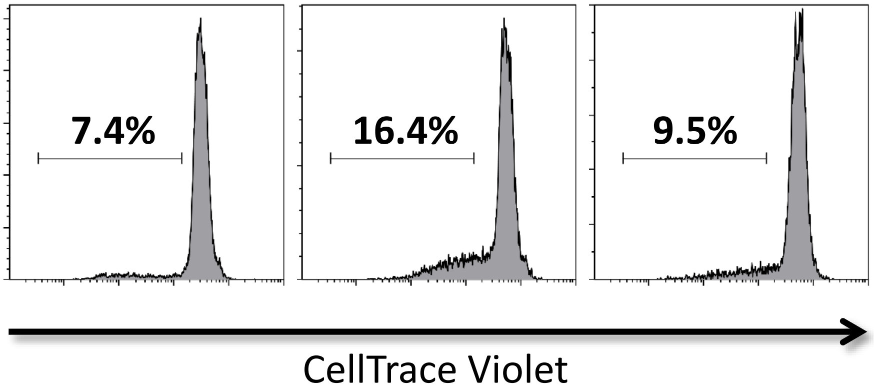

Supplement: Supplementary file 2 — Additional file 2: Fig. S2. Assessing background proliferation resulting from PBMC in the secondary (2nd) MLR. (A) Primary (1st) MLRs were restimulated with autologous PBMC derived from the responder donor to assess background proliferation resulting from non-specific growth promoting effects of PBMCs alone. Shown are representative histogram plots of CellTrace Violet staining of gated responder T-cells. [file 12967_2019_1988_MOESM2_ESM.pdf]
